# Experiences of Inter-Hospital Transfers (IHT) by Patients and Relatives during the COVID-19 Pandemic in France: A Qualitative Study

**DOI:** 10.3390/ijerph20054660

**Published:** 2023-03-06

**Authors:** Nicolas Chauliac, Germain Salome, Juliette Cheucle, Lou Cochennec De Biase, Perrine Galia, Audrey Thomas, Olivier Grimaud, Jean-Marc Philippe, Philippe Vignaud, Nathalie Prieto

**Affiliations:** 1 Centre Régional du Psychotraumatisme Auvergne-Rhône-Alpes, Hôpital Edouard Herriot, Hospices Civils de Lyon, F-69003 Lyon, France; 2 Research on Healthcare Performance (RESHAPE), INSERM U1290 & Université Claude Bernard Lyon 1, Domaine Rockefeller, F-69008 Lyon, France; 3 Faculté de Médecine Lyon-Est, Université Claude Bernard Lyon 1, F-69008 Lyon, France; 4 Univ Rennes, EHESP, CNRS, Inserm, Arènes-UMR 6051, RSMS-U 1309, F-35000 Rennes, France; 5 Direction Générale de la Santé, Ministère de la Santé et de la Prévention, F-75007 Paris, France

**Keywords:** COVID-19, COVID-19 care management, inter-hospital transfer, patients’ experience of care, relatives’ experience of care, mental health

## Abstract

Background: The first wave of the COVID-19 epidemic led to a rapid and unexpected saturation of the French ICU, forcing the health care system to adapt. Among other emergency measures, inter-hospital transfers were carried out. Objective: To assess the psychological experience of patients and their relatives regarding inter-hospital transfers. Methods: Semi-structured interviews were conducted with transferred patients and their relatives. A phenomenological study design was used to examine subjective experiences and their meanings for the participants. Results: The analysis found nine axes pertaining to the experiences of IHT (inter-hospital transfers), grouped in three super-ordinate themes: Information about inter-hospital transfers, differences in patients’ and relatives’ experiences, and host hospital experience. It appears that patients felt little impacted by the transfers, unlike relatives who experienced intense anxiety when the transfer was announced. Good communications between patients and their relatives resulted in a good level of satisfaction regarding their host hospitals. COVID-19 and its somatic consequences seem to have had more psychological impact on the participants than the transfers by themselves. Conclusion: Our results suggest that there are limited current psychological consequences of the IHT implemented during the first wave of COVID-19, although the involvement of patients and their relatives in the organization of the IHT at the time of transfer could further limit them.

## 1. Introduction

In 2019, the emergence of a new coronavirus COVID-19, causing severe acute respiratory syndrome (SARS-COV-2), destabilized the health status and functioning of human societies. In March 2020, the WHO described the COVID-19 epidemic as a pandemic in view of the rapid spread of the virus throughout the world and the worrying discovery of potential systemic and respiratory consequences that could be fatal [1].

In 2020, the French health care system was confronted with the first epidemic wave of COVID-19 (17 March to 11 May 2020). At that time, approximately 5.7% of adults were infected with COVID-19 [2]. As in many European countries during this first wave, the explosion in the number of serious respiratory failures requiring recourse to intensive care units (ICU) caused a shortage of beds in certain regions [3]. In order to adapt, hospitals undertook extensive and rapid restructuring of care, for example, by de-programming less urgent care, increasing the number of beds, hiring additional nurses, importing care equipment such as respirators, and, when these types of measures were not sufficient, undertaking interregional or international transfers of patients to less overburdened hospitals [4]. Inter-hospital transfers (IHT), defined here as transferring ICU patients from one hospital to another, had previously only been carried out on a small scale in France, mainly in military contexts. During the first wave of the pandemic, 644 patients were transferred, mainly from the Grand Est region, and were sent to hospitals in other regions or in the nearest foreign countries such as Germany, Switzerland, or Luxembourg [4]. These first-wave COVID-19 IHTs were unique in their number, the long distances traveled, and the variety of modes of transportation, including ambulances, helicopters, airplanes, boats, and high-speed trains [5].

The physical consequences of these IHTs are the subject of an ongoing retrospective study [5], the results of which have not yet been published. The literature on the psychological effects of IHT is sparse, even outside the specific situation of COVID-19, and often mixes the effects of returning from the ICU to the general ward (termed relocation anxiety or relocation syndrome) with those specifically caused by IHT. In a review of the literature [6], McKinney et al. describe psychological effects ranging from not significant to more severe symptoms such as nightmares and flashbacks. During the COVID-19 crisis, questions concerning transfers focused on the ethical dilemmas of sorting patients, and their capacities to withstand them, leading to the development of tools evaluating the indications for transfer. Although studies are beginning to emerge in the literature concerning the prioritization score for inter-hospital transfer [7] or the experience of caregivers [8], the subjective experience of patients and their relatives has not yet been evaluated to our knowledge. This evaluation seems even more necessary, as the context of care in the ICU is known to cause psychological disorders, both in patients and in their relatives. For example, among patients surviving an intensive care hospitalization, 20% suffer from PTSD [9]. The psychological consequences for relatives are also marked enough for the Society of Critical Care Medicine to define the “Post Intensive Care Syndrome-Family (PICS-F). PICS-F was observed in up to 75% of family members, requiring medication for anxiety or depression in a third of them at patient discharge [9]. It seems that the impact on families goes beyond their personal experience and may compromise their ability to support the patient after discharge [10,11].

In this study, our objective was to explore the experiences of patients and their relatives regarding inter-hospital transfers in intensive care settings during the first phase of the COVID-19 epidemic in France. This question emerged after some of the authors were involved in the national support hotline for COVID-19 patients, which was set up in France shortly after the start of the first wave.

## 2. Materials and Methods

### 2.1. Study Design

Due to the small number of patients with contact information, we chose a qualitative design, which was also able to provide richer and more complex information than a quantitative design. We used interpretative phenomenological analysis (IPA), known for its ability to examine subjective experiences and the meanings given by participants to lived experiences. It is particularly appreciated in the exploration of little-known phenomena [12] and is well suited for the collection of psychic processes and subjective data on the experience of IHT by patients and their relatives. We therefore conducted two parallel analyses on two homogeneous samples: the transferred patients on the one hand and their relatives on the other. The study was conducted in order to fulfill the Consolidated Criteria for Reporting Qualitative Research (COREQ) [13].

### 2.2. Participants

We used convenience sampling with the available data, as less than half the patients were reachable [14]. We then asked every contacted patient if they agreed to give us contact information of a relative. In this way, we recruited two sets of participants: transferred patients and relatives of transferred patients. Participants were identified in the Ministry of Health national registry, SI-VIC (Système d’information pour le suivi des victimes d’attentats et de situations sanitaires exceptionnelles, Direction générale de la santé, Ministère de la santé), which is an information system to record victims of attacks or exceptional health situations.

**Figure 1:**
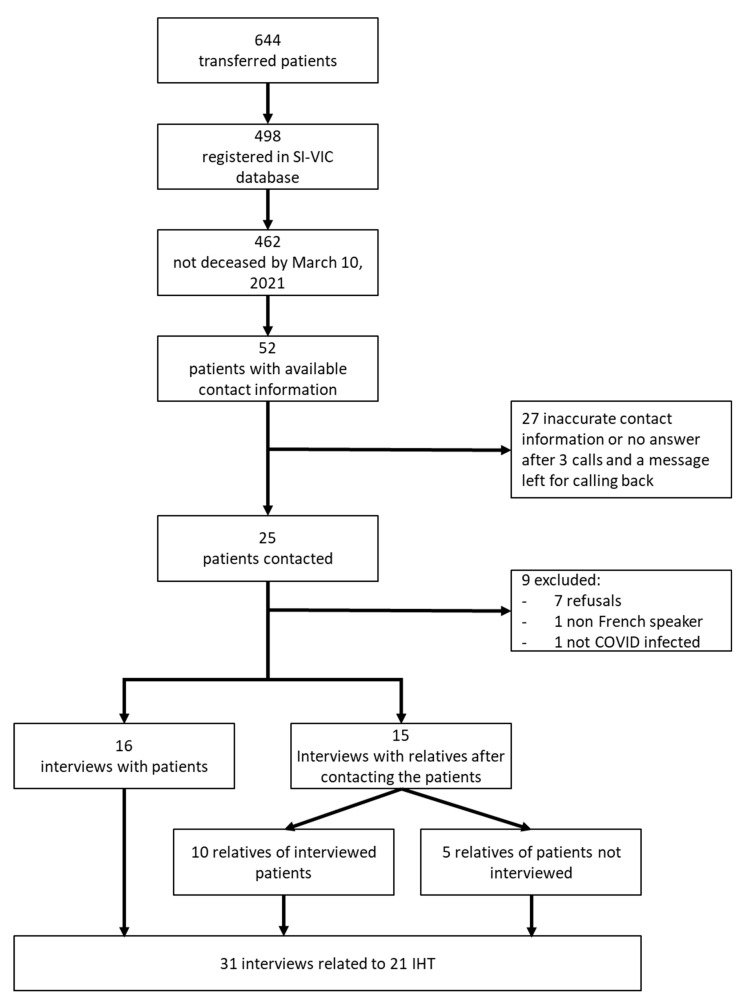
Participants.

Of the more than 600 transfers carried out in France during the first wave, only 498 transferred patients were registered in the SI-VIC database, and 52 were associated with a phone number and not deceased by 10 March, 2021. We tried to contact all of these 52 patients. Twenty-five could be reached and we asked them if they agreed to participate in the study and to give us contact information of one of their relatives. In this way, we set up a sample of 16 transferred patients and 15 relatives of transferred patients, 5 of whom corresponded to relatives of patients not included in the study, and 10 to relatives of included patients (see Figure 1).

### 2.3. Data Collection and Analysis

J.C. and L.C.D.B. conducted the interviews. The interviews with the patients and their relatives were conducted according to the availability of the participants, usually one after the other. A voice-recording device connected to the phone line was used to record interviews. At the beginning of each interview, the interviewer informed the participant about his/her rights to withdraw consent, correct the interview transcription, and about confidentiality and anonymization of the data collected. Participants’ consent and socio-demographic data were recorded at the beginning or the interviews. The semi-structured interviews were based on five open questions related to the subjective experience of the IHT (see Box 1). Questions were similar for the patients and for the relatives, except for question #4. The interviews were then anonymized and transcribed verbatim.

Box 1 **Interview scheme.**

1. What was your personal experience of the transfer?
2. What was the most difficult moment?
3. What was the most positive moment?
4. What are the consequences today on your psychological state, on your daily life, of having been confronted with the inter-hospital transfer (as patient or as relative)?
5. What would be your suggestions for improvement?

Each interview was transcribed and analyzed using NVivo version 1.6.2 software (QSR International, Doncaster, Australia). The data were analyzed according to the guidelines of the interpretative phenomenological method [15], with the following six steps:

1. Multiple readings and re-readings of the transcribed material (G.S., J.C., L.C.D.B., P.G., A.T., N.C.).
2. Line-by-line coding of experiential elements that explain subjective concerns and understandings and allow themes and patterns to emerge (G.S., J.C., L.C.D.B., P.G., and A.T., under the supervision of N.C.). The coding was then reviewed jointly to achieve consensus coding, thus ensuring triangulation.
3. Conceptualizing emerging themes and sub-themes that reflect the psychological essence of the codes resulting from Step 2, while maintaining grounding in lived experience (G.S., J.C., L.C.D.B., under the supervision of N.C.).
4. Searching for links between the emerging themes, assembling the themes, and selecting those relevant to the research question (G.S., J.C., L.C.D.B., under the supervision of N.C.).
5. Switching to the next interview, repeating steps 1 to 4.
6. Looking for patterns across cases: Patterns across cases were searched in two consensus meetings using the transcripts (step 1) and case descriptions (step 4).

### 2.4. Ethics

The study was approved by the ethics committee of the University hospital of Lyon and followed the French regulatory obligations concerning the protection of patients and personal data. The use of contact details from the SI-VIC database to contact patients was approved by the Ministry of Health and the National Health Authority (Direction Générale de la Santé).

## 3. Results

### 3.1. Participants

Participants’ characteristics are shown in Table 1 (for transferred patients) and Table 2 (for relatives of transferred patients). Patients were mostly males, and relatives mostly females. The mean age was 67 years for patients and 55.5 years for relatives. Almost two-thirds of the patients and half of the relatives had a low level of education (below upper secondary level). Two-thirds of patients and two-thirds of relatives had a low to moderate income. Relatives were mainly spouses or children of the patients.

### 3.2. Interpretative Phenomenological Analysis

**Figure 2:**
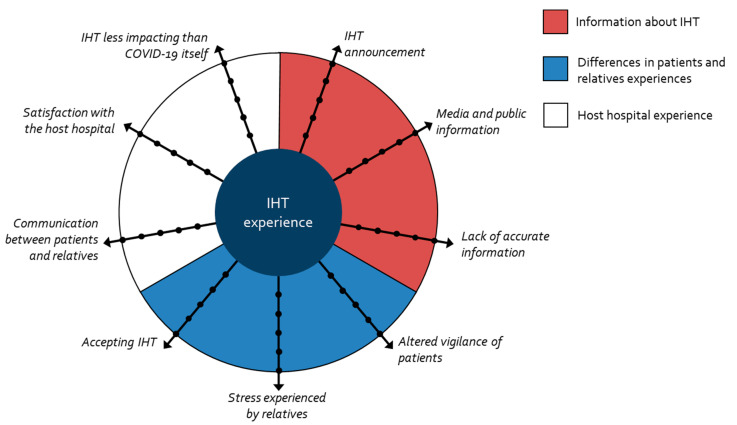
Interpretative phenomenological analysis: themes and sub-themes.

Three super-ordinate themes emerged from the analysis, sustaining the understanding of the experiences of patients and their relatives: the announcement of the transfer, the difference in perception of the transfer between patients and their relatives, and the experience of care in the receiving hospital. Through these main themes, the quality of communication throughout the transfer appears to be a preponderant factor in the way it was experienced. These three themes were associated with nine axes: IHT announcement, media and public information, lack of accurate information, altered vigilance of patients, stress experienced by relatives, accepting IHT, communication between patients and relatives, satisfaction with the host hospital, and IHT less impacting than the COVID-19 itself, as shown in Figure 2.

#### 3.2.1. Information about IHT

Information about the transfer was often described as inadequate, whether it was the late announcement of the transfer (or sometimes the absence of any announcement), or the lack of information about the modalities or the destination. However, relatives were sometimes able to picture these transfers, based on information in the media. The sub-themes that emerged to describe this experience are detailed below.

##### IHT Announcement

The way in which the transfer was announced had a significant impact on the level of anxiety and the perception of the transfer by the relatives. Relatives who were informed of the transfer beforehand perceived it more positively and could see it as a sign of their relative’s clinical stability.

*“He told me that my husband was lucky in his misfortune that he was already intubated, already in care and stable. So he could be transferred while they were expecting the worst for the next patients. So I was obviously reassured that he had left.”*

(R9)

Conversely, relatives who found out about the transfer late, sometimes without contact with a physician, may have experienced anxiety or confusion, as well as a sense that the transfer was related to a serious clinical deterioration or poor prognosis.

*“When you learn that he is going to Marseille, when there is nothing to suggest that… you are already asking yourself questions, so in the evening, of course, we asked ourselves questions and my daughter, the first thing she thought was: ‘Mum, if he is transferred, he’s not well at all.’”*

(R14)

##### Media and Public Information

Depending on their exposure to media coverage of hospital congestion, participants received the transfer announcement in different ways. Those who were aware that local hospitals were overloaded welcomed the transfer as a chance for patients to receive care outside the severe epidemic context of their region, and possibly to receive better quality care. Their relief was sometimes accompanied by fears that the patient would be neglected locally.

*“Then we watched the news to see if they had found a treatment and all that, and we heard that they might have to choose [which patients to care for]. Because with his 110 kilos, his diabetes, his hypertension, we knew that if there was a choice to be made it wouldn’t be him.”*

(R9)

*“…they know what they’re doing, he’ll be taken care of in a team, they’ll have more time. Because I knew that it was overloaded in Colmar. We were the first region to be affected. And so I knew that here they were overworked and I told myself that over there they would take more time. In the end, I was perhaps, I almost would say, rather relieved that he went elsewhere. Now he’s better taken care of…”*

(R13)

*“And they explained everything to me. No, it was precisely because he was getting better and we had to give way to other people who were arriving because there were no more beds. So… my surprise lasted a few minutes, but afterwards, they explained to me very well why. Then, I was following the news, so I understood very well afterwards, I had no problem with it.”*

(R11)

##### Lack of Accurate Information

Some relatives were worried about the lack of visibility of the conditions and destination of the transfer and felt that there was a lack of coordination between the sending and receiving hospitals due to the urgency of the transfers.

*“The most difficult thing was the transfer itself, there’s no doubt about it, when they called us, because we didn’t know exactly where she was going, it was only once she was in the helicopter that they told us where she was going.”*

(R2)

*“…the lack of information, especially about the fact that… Yes, I was told that she had been in a helicopter but they couldn’t tell me where she had landed… That’s what made me a little bit angry. But let’s say I knew she had arrived in another hospital, but I didn’t know which one…”*

(R5)

*“So my daughter, the next day, when the transfer was made, she struggled, she called the hospitals in Marseille to find out which hospital he had been transferred to. I mean, it was… She fought, she managed to find out where he was, she got some answers and that reassured us.”*

(R14)

Some relatives insist that their concern was more related to the lack of information about the transfer than to the transfer itself.

*“The way of communicating, taking time… It’s not obvious for the nursing staff, the doctors but… it’s a trauma as much for the family as for the patients, and when there is no information, it’s even worse.”*

(R9)

#### 3.2.2. Differences in Patients and Relatives Experiences

This second theme relating to the experience of inter-hospital transfer highlights the significant gap between the experiences of patients and those of their relatives.

##### Altered Vigilance of Patients

Almost all patients were transferred while in a coma. They also showed confusion when they woke up. Therefore, they were not immediately aware that they had been transferred to another hospital.

*“Yes, it was explained to me, but I didn’t understand everything straight away because it took me a while to be coherent in fact, even after waking up. It took me a few days to be able to…there was a doctor who spoke French, and so he explained to me roughly what had happened, but it was a bit vague.”*

(P2)

The patients describe this transfer as not very disturbing for them: most of them have no memory of it and have reconstructed it from the elements reported by their relatives.

*“My son told me afterwards anyway, the twists and turns…but otherwise, I don’t remember anything at all. Everyone says to me, ‘Look, we’ve been worried sick about you’. Well, I in a coma…I didn’t think that at all because I didn’t sort of live it.”*

(P8)

##### Stress Experienced by Relatives

In contrast to the patients, the relatives were often more concerned about the patients’ physical condition and chances of survival, sometimes beyond the question of transfer.

*“…for me the most important thing is that he gets away with it, here or elsewhere.”*

(R13)

*“The most difficult part was whether he would return from Germany alive or dead.”*

(R3)

In addition, cross-border transfers may have increased anxieties related to the language barrier and difficulties in interacting with health care teams.

*“The problem I had was that I very often came across someone different and it was in a part of Switzerland where they speak Swiss German and they didn’t speak English, they didn’t speak French, and I don’t speak Swiss German. And so I had one of the doctors who spoke French, I had one or two doctors who spoke English, but often we didn’t understand each other very well”.*

(R9)

*- “ [Interviewer] What was the most difficult moment for you?”*

*- “Not to understand what state she was in, because of the language barrier.”*

(R1)

Relatives also had to deal with several practical issues, adding to their worries, such as the question of paying for care abroad, having to organize the transfer back to the hospital of departure, or the anticipation of a death at a great distance.

*“The hospital in Switzerland contacted me and said: we need the [credit] card to pay for the care… My mother, she had had a complication. For a while, France didn’t really agreed to pay for the complication, but in fact it’s a complication of COVID-19. So I got a little upset and they covered the costs. In the end, I hardly had to pay anything… But it’s horrible to think about that when she might be dying…”*

*“On the other hand, the return was difficult because the host hospital did not want to pay for the return. He had to return by his own means, but he was not yet able to stand up well. But then, it was the doctor there who had to get angry because the hospital didn’t want to pay for the return.”*

##### Accepting IHT

Both patients and family members retrospectively consider the IHT to be a good decision.

*“I think it’s a good decision that they made so that there are other lives that are saved.”*

(P3)

*“It was rather a chance because in Strasbourg they couldn’t take care of the patients anymore, they were overwhelmed.”*

(R11)

However, the relatives mentioned that prior information and the possibility of a phone call on arrival at the receiving hospital would reduce their anxiety.

*- “ [Interviewer] What could be improved regarding IHT?”*

*- “To give people a bit more notice and information so that they know where their loved ones are going and which hospital they are going to, to give them more… how should I put it… answers… and the phone number, you know.”*

(R4)

For relatives, the inability to visit patients in the ICU was not seen as an additional obstacle in the context of the general restriction of access to the ICU for relatives, despite the fear that patients might die without being able to see them again.

*“Knowing that in this case he was, in fact, in a coma. So, for me, whether he was here or elsewhere, it didn’t matter much because we weren’t allowed visits anyway.”*

(R12)

#### 3.2.3. Host Hospital Experience

This theme describes the experience of care in a remote hospital, highlighting the importance of communication between patients and relatives, an overall satisfaction with the host hospital, and a relativization of the consequences of IHT compared to those of COVID-19.

##### Communication between Patients and Relatives

Remoteness was reported as having an impact by patients and relatives:

*- “ [Interviewer] Was there a moment that was particularly difficult?”*

*- “After I woke up. That’s it, that’s the most… I was in a room, all alone, and I was like abandoned.”*

(P6)

*“Everything is done over the phone, distantly. The first video where we saw him, we made like a big mistake, but we did it because we all wanted to. There were 15 of us in a 9 square-meter kitchen, to see him, you see… That’s the hardest thing to live with.”*

(R10)

The subjective experience and anxiety generated by the IHT was reported to be less burdensome when direct communication between patients and relatives was possible, particularly by means of letters or by video calls. Relatives and patients mentioned the improvement in their psychological experience that regular contacts had brought.

*“When I woke up, they gave me some mail. I had a letter for my birthday, that my German friend sent me and he said: when you read this birthday card, then you’ll be awake and you’ll know that you’re in Germany… And that made me really happy.”*

(P3)

*“And from the moment he managed to reach us by phone and say a few words to us by phone, that was the most positive moment.”*

(R8)

##### Satisfaction with the Host Hospital

Patients could report satisfaction with the care provided by the host hospital, although their levels of alertness and lucidity were often not fully restored during hospitalization.

*“I didn’t understand why I was there and nobody ever explained it to me…maybe I never asked for anything as I’m a bit…Well. Anyway, I was very well looked after there.”*

(P4)

*“I was in a ward where they were great, very nice, very good, they looked after me really well.”*

(P16)

Communication and accessibility for the relatives appear to play an important role regarding the host hospital.

*“… I was happy because, as he was confused, I had sent a photo, I had sent photos of the family at home, and to tell him what year it was. So that he could have them as a reference. My father had received them. He told me that it had been very important for him.”*

(R3)

##### IHT Less Impacting than COVID-19 Itself

Among patients, the experience of the symptoms of COVID-19 are at the forefront, overshadowing the consequences of the IHT.

*“…it’s maybe not even so much the transfer, but COVID, it’s the most traumatic, the most striking. It’s not even the transfer since I wasn’t conscious.”*

(P3)

*“The important thing is that he makes it, here or elsewhere”.*

(R13)

Patients could report psychiatric symptoms in the first few weeks after being extubated, such as panic attacks, anxiety, and intrusive memories. However, these symptoms appear similar to those that are well described in the aftermath of hospitalization in an ICU.

*“I knew I was tied up, so at bed level, and I always had this desire to get free and probably I was ripping out the wires, the IVs and all the system that was hooked up to me. So they had to tie me up for that. So I just have a few flashes…”*

(P3)

## 4. Discussion

### 4.1. Main Results and Relatvie Existing Literature

To our knowledge, our study is the first to focus on the experiences of emergency IHT due to COVID-19 hospital overload. In this qualitative study, we derived nine axes from patients’ and relatives’ interviews regarding their experiences with IHT, grouped into three clusters: information about IHT, differences in patients’ and relatives’ experiences, and host hospital experience.

All participants emphasized the importance of access to accurate and timely information about IHT. Indeed, IHT during the first wave of COVID-19 occurred in a context of emergency and did not allow for anticipated or shared decisions with patients. The anxiety, helplessness, or anger experienced by relatives when informed of transfers are also described in the literature about ICU admission; relatives who felt information was incomplete about their loved ones have shown higher rate of Post Intensive Care Syndrome–Family (PICS-F) [16]. Informing relatives about the possibility of a transfer to an ICU or an IHT may reduce the stress caused by the sudden discovery of the need for transfer. This interpretation is supported by the results of a study using psychometric scales to compare the experiences of families whose relatives were admitted to the ICU for acute respiratory distress, either directly from the emergency department or operating room of the same hospital, or transferred from the ICU of another hospital [17]. Scores of psychological distress and posttraumatic stress increased when the patients were transferred. These results may be explained by the level of stress caused by the announcement of a transfer. Clear and anticipated information before transfer in and out of the ICU has been shown to reduce patients’ feelings of uncertainty, hopelessness, and vulnerability and perceptions of loss of security [11]. It is also interesting to note that IHT were viewed differently depending on the pre-existing knowledge about this type of transfer and the congestion situation of the local hospitals. Thus, general information about the transfers through the local media could be used as a first information tool to inform relatives about the possibility of IHTs.

Patients’ and relatives’ experiences differ, mainly because patients were in a coma before and for a few days after transport, often followed by symptoms of confusion or delirium, which resulted in the absence of memories of transport and relatively few memories of care at the receiving hospital. In fact, the patients interviewed did not report any symptoms that could indicate a transfer syndrome. This phenomenon, often described in the literature as one of the psychological consequences of leaving the ICU, whether within or between hospitals, is characterized in particular by physical symptoms, great anxiety, and feelings of insecurity and abandonment when leaving the ICU [6]. For their part, relatives had to deal with many practical issues that added to the stress of IHT and the daily worries about a distant death. Although, retrospectively, relatives and patients show a good acceptance of IHT, it is important to note that, in the particular context of the first wave of COVID-19, almost all visits to patients in hospitals were prohibited, which greatly mitigated the impact for relatives of having a loved one transferred in a distant hospital. Had ICUs been accessible to relatives, as recommended by good practice [18], relatives may have experienced greater frustration at not being able to visit their loved one, or the inconvenience of long journeys to do so.

The host hospital experience encompasses three axes: communication between patients and their relatives, overall satisfaction with the care provided in the host hospital, and a general feeling that the symptoms and consequences of COVID-19 had a much greater impact than the IHT. The improvement in the experience of care due to the availability of the host hospital staff to the relatives and the use of communication tools when the patient’s condition allowed (letter, telephone, video) is consistent with the widely described negative consequences for families of a ban on ICU visits and more frequent symptoms of post-traumatic stress, complicated grief, and anxiety [16,19,20,21], but also with a quantitative study that showed the importance of communication in satisfaction with ICU care after IHT [17]. A large part of the participants’ discourse repositioned IHT in an emergency context, where all means to avoid deterioration of the patient are legitimate. The issue of transfer and remote care then takes second place to that of the risks and after-effects associated with COVID-19, confirming the results of a study that showed that the psychological consequences of these transfers for relatives are mainly related to the severity of the patient’s illness rather than to the transfer itself [17]. This could also be related to natural resilience over time, which influence could be evaluated in our study.

### 4.2. Limitations of the Study

The study did not include relatives of patients who had died in the aftermath of the transfer (26 out of 498 in the SI-VIC database). Their experiences may have modified the results, as 5 to 45% persons whose relative died in ICU meet criteria for complicated grief [9]. In addition, confounding factors such as memory bias, as well as the natural process of resilience regarding the psychological consequences of IHT, may be present with respect to the 18 months that elapsed between the transfers and the interviews. However, this long delay may also be seen as a filter that retained only the most important and salient experiences. Finally, our sample was very limited because the SI-VIC data on contact information were scarce and sometimes erroneous.

### 4.3. Perspectives

The nine axes identified in our study could be used as a metric to capture the experience of patients and families with a simple satisfaction questionnaire, as suggested in Table 3. As scores can be assigned to each axis, this could be used in a quantitative study to assess the relationship between the score of each axis and the overall IHT experience, or to refine the correlation between the axes. In addition, it could be used to explore non-COVID-19 IHT experiences.

## 5. Conclusions

The psychological impact of the transfer on the patients and their relatives, as assessed 18 months after the COVID-19 first wave, did not seem to be very marked, although the conditions of announcement and organization of the transfers could trigger stressful situations for the relatives. The transfer itself was well tolerated and the care at the host hospital was considered very satisfactory. The IHT was often perceived as an opportunity to receive appropriate care in regions less affected by the COVID-19 epidemic. Clinically, our study highlights that the involvement of patients and their relatives in the organization of an IHT could limit the negative psychological consequences for patients and their relatives by reducing the stress experienced.

## Figures and Tables

**Table 1:** Patients’ characteristics.

Interview Number	Sex	Age at IHT Date (y.o.)	Country of Arrival	Education Level	Annual Income (k€)
P1	F	68	Switzerland	tertiary education	<15
P2	M	54	Germany	below upper-secondary	25–50
P3	M	61	Germany	tertiary education	25–50
P4	F	67	France	below upper-secondary	25–50
P5	M	68	France	below upper-secondary	<15
P6	M	69	Germany	below upper-secondary	15–25
P7	M	57	Germany	tertiary education	25–50
P8	F	75	France	tertiary education	15–25
P9	M	63	France	below upper-secondary	15–25
P10	M	76	France	Does not wish to reply	Does not wish to reply
P11	M	74	France	Does not wish to reply	Does not wish to reply
P12	M	60	France	below upper-secondary	<15
P13	M	63	France	below upper-secondary	15–25
P14	M	65	France	upper secondary	15–25
P15	M	75	France	below upper-secondary	Does not wish to reply
P16	M	77	France	below upper-secondary	<15

**Table 2:** Relative characteristics.

Interview Number	Sex	Age at IHT Date (y.o.)	Relationship with the Patient	Education Level	Annual Income (k€)
R1	F	35	Daughter	tertiary education	>50
R2	M	68	Husband	below upper-secondary	Does not wish to reply
R3	F	30	Daughter	tertiary education	15–25
R4	F	66	Wife	below upper-secondary	15–25
R5	M	57	Husband	below upper-secondary	25–50
R6	F	65	Wife	below upper-secondary	<15
R7	F	72	Wife	below upper-secondary	Does not wish to reply
R8	F	46	Daughter	tertiary education	15–25
R9	F	52	Wife	upper-secondary	25–50
R10	M	45	Sound	upper-secondary	15–25
R11	F	71	Wife	tertiary education	15–25
R12	F	59	Wife	below upper-secondary	Does not wish to reply
R13	M	34	Son	below upper-secondary	15–25
R14	F	59	Wife	upper secondary	<15
R15	F	74	Wife	tertiary education	Does not wish to reply

**Table 3:** Questionnaire for the collection of IHT experiences.

		1 Low	2	3	4	5	6	7	8	9	10 High
Information about IHT	Are you satisfied with the conditions of information about the transfer?										
	Rate the clarity of the information you received about the transfer										
	What was your level of information about your local hospital’s ability to admit your family member?										
Differences in patients and relatives experiences	What was your or your relative’s average level of lucidity during the transfer and hospitalization?										
	What level of general stress did you experience?										
	Do you feel that the transfer was appropriate for the situation?										
Host hospital experience	Are you satisfied with the means used to communicate with your loved one?										
	Are you satisfied with the care provided by the host hospital?										
	Would you say that the transfer was much less significant than the issue of your loved one’s health condition?										

## Data Availability

The data presented in this study are available on request from the corresponding author. The data are not publicly available because the interview transcriptions contain information that could lead to identifying the participants.
